# Hematological Malignancies Escape from NK Cell Innate Immune Surveillance: Mechanisms and Therapeutic Implications

**DOI:** 10.1155/2012/421702

**Published:** 2012-08-01

**Authors:** Laure Farnault, Carole Sanchez, Céline Baier, Thérèse Le Treut, Régis T. Costello

**Affiliations:** ^1^UMR1090, Aix-Marseille Université, TAGC, 13008 Marseille, France; ^2^Service d'Hématologie, CHU La Conception, AP-HM, 13005 Marseille, France; ^3^Laboratoire d'Hématologie, CHU Nord, AP-HM, 13015 Marseille, France

## Abstract

Hematological malignancies treatment improved over the last years resulting in increased achievement of complete or partial remission, but unfortunately high relapse rates are still observed. Therefore, sustainment of long-term remission is crucial. Immune system has a key role in tumor surveillance. Natural killer (NK) cells, at the frontier of innate and adaptive immune system, have a central role in tumor cells surveillance as demonstrated in the setting of allogenic stem cell transplantation. Nevertheless, tumor cells develop various mechanisms to escape from NK cells innate immune pressure. Abnormal NK cytolytic functions have been described in nearly all hematological malignancies. We present here various mechanisms involved in the escape of hematological malignancies from NK cells surveillance: NK cells quantitative deficiency and NK cell qualitative deficiency by increased inhibition signaling or decreased activating stimuli. A challenge of immunotherapy is to restore an efficient antitumor response. A combination of classical therapy plus immune modulation strategies will soon become a standard of care for hematological malignancies.

## 1. Introduction

Hematological malignancies are cancers that affect blood, bone marrow, and lymph nodes, thus maintaining a slight contact with immune system cells. Although complete remission (CR) rates have increased over the last years, the high incidence of relapse impairs long-term prognosis. While achievement of CR mainly relies on high-dose chemotherapy, long-term control involves maintenance protocols with daily, weekly, or monthly low dose of chemotherapy prolonged for some months or years, as well as the induction or restoration of immune pressure against minimal residual disease (MRD).

Natural killer (NK) cells play a key role in the immune antitumor response as demonstrated by the low relapse rate obtained in allogenic stem cell transplantation when there is a mismatch of NK inhibitory receptors between the host and the graft [1]. NK cells are lymphocytes that have cytotoxic properties and cytokine-production capacities. They are innate immune-effector cells that recognize and kill transformed cells (i.e., tumor cells and virus-infected cells). NK cells also have immune regulatory functions thanks to cytokine and chemokine secretion, which favor the development of a T-helper cell (TH1) response [2].

The role of NK cells in immune monitoring of tumors is essential mainly due to their nonhuman leukocyte antigen (HLA) restricted effect, as the absence or abnormal expression of HLA molecules induces NK-cell cytotoxicity (the so-called “missing self” hypothesis of Karre [3]). Indeed, as a consequence of antigen-specific immune pressure by T-cells, tumor cells with downregulated HLA class I molecules are progressively selected and thus could become targets for NK cytotoxicity.

Phenotypically, NK cells are CD3—lymphocytes that express CD56 and/or CD16 at different levels. CD16 is the Fc*γ*RIIIa receptor that can mediate antibody-dependent cell-mediated cytotoxicity (ADCC). When a NK cell has recognized its target cell, it kills the target cell by secreting cytotoxic granules (perforin, granzyme, and granulysin), *via* interaction with the tumor-necrosis factor family (Fas/FasL) or even by cytokine secretion.

NK cells express a large panel of cell-surface activating and inhibitory receptors that recognize ligands on potential target cells. The balance between the signals delivered by activating and inhibitory receptors determines whether or not NK cells kill a target cell and secrete cytokines [4, 5]. Thus, a “dynamic equilibrium concept” between these receptors controls NK cell activation.

Activating receptors are mainly represented by the natural cytotoxicity receptors (NCR) NKp30/NCR3, NKp44/NCR2, NKp46/NCR1, and NKG2D, but also by coreceptors such as 2B4/CD244 or NTBA. Activating receptors have various ligands (see review [6]), such as the stress-induced molecules HLA class-I chain-related A (MICA) and MICB, which activates cytotoxicity in NK cells through their ligation to NKG2D, leading to the destruction of the target cell. Conversely, NK cell cytotoxicity is downregulated by the engagement of HLA-specific inhibitory receptors (killer immunoglobulin-like receptors (KIR) and CD94/NKG2A/B heterodimers), thus protecting normal cells. The recognition of normal HLA class I molecules on target cells downregulates NK cytotoxicity. 

Defects in NK-cell cytotoxicity have been observed in all hematological malignancies [7–10]. The escape of hematological malignancies from NK cell immunity can be explained by general mechanisms that are common to all immune-effector cells (see review [4]), that is, saturation of the immune system by rapid growth of the tumor, inaccessibility of the tumor because of deficient vascularization, but also by dysfunction of the immune system that could be restored by immune modulatory intervention. In this paper, we focus on tumor escape from NK cells surveillance by evocating successively various mechanisms involved (summarized in Table 1): the quantitative deficiency of NK cells, their qualitative impairments caused by increased inhibition, or decreased activation signaling. We then examine the key role of cytokine environments in tumor immune escape from NK cells. Finally, we briefly evocate various therapeutic means to enhance NK cell control of hematological malignancies.

## 2. Tumor Escape from NK Cell Surveillance: **** Role of NK Cells Effectors' Quantitative ****Deficiency

Quantitative deficiency is the first mechanism in myelodysplastic syndrome (MDS) to explain how tumors escape from innate immune surveillance. MDS is a preleukemic syndrome characterized by clonal hematopoietic stem-cell disorders and peripheral cytopenia. In 1984, Kerndrup et al., noticed that the decreased NK activity seen in patients with MDS was caused by a decreased number of circulating NK cells [5]. In 1994, Yokose et al., correlated the decreased absolute number of CD3−CD16+ and CD3−CD56+ cells, in patients that had a high risk of MDS, with an increased plasmatic level of sIL-2R [11]. The authors hypothesized that plasma sIL-2R is produced by malignant MDS cells in the bone marrow and could impair IL-2 stimulated growth of NK cells. Nonetheless, an increased absolute number of NK cells can be associated with impaired cytotoxicity (Sanchez et al., personal data). Thus, in most hematological malignancies, qualitative impairment of the capacity of NK cytotoxic seems more important for tumor escape than quantitative defects.

## 3. Tumor Escape from NK Cell Surveillance: **** Role of NK Cells Effectors' Qualitative ****Deficiency

### 3.1. NK Cell Qualitative Deficiency by Increased Inhibition of NK Cell Cytotoxicity

Decreased expression of HLA class I molecules is a way for tumor cells to escape specific T-cell surveillance [35, 36]. Interestingly, the downregulation of HLA class I may allow NK cell targeting of tumor cells. Nevertheless, some reports from acute myeloid leukemia (AML) show a normal expression of HLA class I that inhibits the potential action of NK cells and leads to NK cell anergy [12]. Upregulation of HLA-A, -C, and -E molecule surface expression was demonstrated in a drug-resistant leukemic cell line that was also resistant to NK cell cytotoxicity [13]. Demanet et al. showed, in leukemic cells from 24 patients with acute lymphoblastic leukemia (ALL) and chronic lymphocytic leukemia (CLL), selective downregulation of HLA-A and HLA-Bw6 associated with HLA-Bw4 preservation, which provided an escape mechanism not only from T-cells but also from NK cell surveillance [14].

Similarly, in CLL cells, Maki et al. have demonstrated and increased the expression of tolerogenic HLA-G1, a class I molecule that engages NK cell inhibitory molecules. Blockade of HLA-G1 with a specific antibody in CLL samples increased their susceptibility to NK-mediated killing, demonstrating that HLA-G1 participates in protecting CLL cells from NK-mediated killing [15]. Beside leukemia, upregulation of HLA class I has been also described in multiple myeloma and lymphoma [16, 17]. High expression of HLA-I molecules is observed in late-stage multiple myeloma (MM) plasma cells, and confers protection from NK lysis, even though NK cells efficiently kill early-stage tumoral plasma cells in a NCR- and NKG2D-dependent pathways [17].

### 3.2. NK Cell Qualitative Deficiency by Impaired Activation of NK Cells

In AML, the downregulation of NCRs NKp30/NCR3 and NKp46/NCR1 is associated with decreased NK cell cytotoxicity [8, 18]. In sharp contrast with healthy donors, in most patients with AML, the majority of NK cells display low NCR surface density (NCR^dull^). This phenotype correlates with their weak cytolytic activity against autologous leukemic cells, which cannot be reversed by monoclonal antibody-mediated disruption of HLA class I/KIR-receptor interaction. This phenotype is partially or totally reversed when patients achieve CR, suggesting that this phenotype is probably acquired by direct contact between leukemia blasts and NK cells [19]. In addition, NK cell activity correlates positively with the relapse-free survival of patients with AML [7]. In CLL, although no difference is observed regarding NCR expression between patients and age-matched healthy controls, decreased NCR expression correlates with a poor prognosis [21]. Another mechanism that is pivotal to the immune escape of AML blasts, is the downregulation of ligands relevant in NCR-mediated target-cell recognition [8]. Together, insufficient activation, caused by defects in NCR expression and deficient expression of their ligands on leukemic target cells, may represent a potent method for tumor immune evasion. These abnormalities, when present at the diagnosis of leukemia, may also be related to a worse outcome [19]. 

Another activating receptor, NKG2D, has a crucial role in NK cell activation. The NKG2D ligands, MICA, and MICB [37], are expressed on half of AML blasts [38]. Leukemia cells that express MICA are lysed *in vitro* by NK cells via NKG2D engagement with the sera of patients, but not of healthy donors as the latter contain elevated levels of seric soluble MICA (sMICA), which impairs NKG2D-mediated immune surveillance of leukemia by triggering internalization of surface NKG2D [39]. In large granular lymphocyte (LGL) leukemia, the impaired cytolytic function of NK cells is associated with reduced expression of NKG2D, which correlates with disease progression [22]. Abnormally high levels of sMICA and weak expression of NKG2D on NK cells have also been reported in chronic myeloid leukemia (CML) [40]. Interestingly, these abnormalities are reversed by imatinib mesylate therapy. In this setting, the level of MICA expression is under the posttranscriptional control of BCR/ABL tyrosine-kinase activity [40]. Similarly, MM patients have high levels of sMICA whereas tumor cells express low levels of MICA [24]. In contrast, in monoclonal gammopathy of undetermined significance (MGUS), a common disorder of aging and a precursor lesion to MM, tumor cells express high levels of MICA whereas low levels of sMICA are detected in peripheral blood. These data suggest that alterations of the NKG2D pathway are associated with progression from MGUS to MM [24]. Alteration of the NKG2D pathway is also observed in CLL and could, in part, be responsible for the defective NK cytotoxicity observed [15]. CLL leukemic cells do not express MICA- or UL-16-binding proteins (ULBPs) (excepted a very low expression of ULB3) [23]. Similarly, NK-resistant B-ALLs cells do not express MICA, MICB [25], or ULBPs (or only at very low levels) [41].

Recently, Weiss-Steider et al. demonstrated that myelomonocytic leukemia cells produce and secrete MICA and MICB, but also express the receptor NKG2D, resulting in an *in situ* depletion of stress signals, thus avoiding activation of NK cells [26]. Moreover, MICA and MICB appeared to act as a tumor-growth factor resulting in strong tumor proliferation in dose-dependent induction [26].

In addition to the downregulation of NCRs and NKG2D, downregulation of the NK-cell activating receptors or the co-receptor DNAM-1, 2B4/CD244 and CD94/NKG2C have been described in AML [38]. Similar to the NKG2D pathway, chronic exposure of these activating molecules to their ligands may be responsible for their downregulation [27]. This has been demonstrated for DNAM-1 since an inverse correlation between DNAM-1-ligand expression on leukemic blasts and DNAM-1 expression on NK cells has been found. Furthermore, the culture of NK cells from healthy donors with leukemic blasts that express DNAM-1 ligands induce DNAM-1 downregulation at the NK cell surface [27]. NK cell costimulatory molecule 2B4/CD244 has a natural ligand that is CD48, a molecule that is highly expressed in hematological malignancy cells [42]. In MM, despite normal NCR and NKG2D expression [17, 28, 29], there is drastic downregulation of CD16 and 2B4/CD244, which leads to decreased ADCC in MM [28, 29]. The downregulation of CD16 can be explain by the continuous exposure to circulating monoclonal immunoglobulin.

### 3.3. NK Cell Qualitative Deficiency by Impaired Differentiation Signaling

Altered NK differentiation from CML CD34+ progenitors is linked to their constitutive production of bioactive IL-15, which does not lead to NK cell differentiation [43]. This has been directly attributed to the *BCR*-*ABL* mutation, because the addition of BCR-ABL-transfected stem cells suppresses the differentiation of autologous normal cord blood CD34+CD38− [30]. Impairment of the signaling pathway is also suspected in polycythemia vera (PV), an MP disorder associated with an acquired activated mutation of the tyrosin kinase JAK2. Patients with PV have an increased percentage and absolute number of NK cells with impaired cytotoxic functions, not caused by variation in inhibitory or activating receptors (Sanchez et al., personal data). The NK cytotoxic defect may be related to the direct or indirect impairment of cytotoxicity signaling pathways, putatively linked to JAK2 mutation. We are currently testing this hypothesis via the transcriptomic study of NK cells taken from PV patients (Baier et al., personal data).

## 4. Tumor Escape from NK Cell Surveillance: The Pivotal Role of Cytokines

Several cytokines are known to inhibit NK cell activation. In 1992, Verhoef et al. suggested that elevated and circulating levels of tumor-necrosis factor (TNF) impairs NK cytotoxicity in MDS [31]. Similarly, the high levels of PDGF, detected in myeloproliferative syndrome (MPS) could impair NK cell cytotoxicity [32]. TGF-*β* has also been implied in the inhibition of NK cell function following chronic interaction with tumor cells [33]. Indeed TGF-*β* antagonizes IL-15, which induces proliferation and gene expression associated with NK cell activation, resulting in inhibition of both NK cell activating receptor molecules and components of the cytotoxic apparatus [33]. Impaired NK cell function as been also linked to a decrease production of NK cell activating cytokine such as IL-1, interferon-gamma and IL-2 in the peripheral blood mononuclear cells of AML and ALL patients [34].

## 5. Therapeutics Implications “from Bench to ****Bedside”

As chemotherapy can disrupt potentially competent immune-surveillance mechanisms, thus favoring a relapse via induced immune suppression, the necessity of developing immunotherapy that enhances immune surveillance after chemotherapy is discontinued is admitted [44]. Markasz et al. have characterized the effect of 28 frequently used chemotherapeutic agents on the capacity of NK cells to kill tumor cells [45]. Although most chemotherapy drugs quantitatively decrease NK cell counts, some inhibit NK-cell activity whereas others enhance NK cells ability to kill tumors cells [45].

The greatest revelation of NK cells role in tumor control has been shown in allogenic transplantation. In 2002, Ruggeri et al. demonstrated a favorable prognosis for AML patients who were recipients of a haploidentical allogenic transplant with a NK HLA-specific receptor mismatch [46]: this enhanced the antileukemic graft reactivity. Interestingly, NK cell mismatch provides a graft-versus-leukemia (GvL) effect that is devoid of the deleterious graft-versus-host (GvH) reaction. This is because host cells, apart from leukemia cells, do not express activating ligands for NK cell cytotoxicity [47, 48]. Conversely, the benefit of NK-cell alloreactivity after unrelated cord-blood stem-cell transplantation remains unclear and is controversial [49]. Miller et al. were the first to infuse NK cells from haploidentical origins into AML recipients in a nontransplant setting [50]. Haploidentical NK cells can persist and expand *in vivo* if patients receive intensive immunosuppression regimens before NK cell infusion [50]. Successful transfer of alloreactive haploidentical KIR ligand-mismatched NK cells was performed in 13 elderly patients after administration of a combined immunosuppressive therapy of fludarabine plus cyclophosphamide, followed by subcutaneous administration of IL-2 for 2 weeks. Donor *versus* recipient alloreactive NK cells were shown to kill recipient leukemia cells. This phase I study demonstrates that infusion of purified NK cells is feasible and safe [51]. A phase II study, using a greater dose of NK cells and multiple infusions is being currently conducted. Similarly, a pilot study on transfer of alloreactive haploidentical KIR ligand-mismatched NK cells has been performed in children with AML after achievement of CR. The study gave encouraging results, and has allowed commencement of a phase II trial as consolidation therapy in children with AML [52]. In MM myeloma, infusion of haploidentical KIR-ligand mismatched NK cells has been proposed in relapsed patients in the setting of autologous stem-cell transplantation [53].

Since allogeneic transplantation raises several problems regarding high morbidity and mortality rates, manipulation of autologous NK cells rises increasing interest. The IL-15 supports large-scale expansion of NK cells by preserving activating-receptor expression [54]. *In vivo*, the TGF-*β* blockade, by both anti-TGF antibodies and a small molecule inhibitor of TGF-*β* signaling, is an interesting way to suppress the NK-cytotoxicity inhibition caused by TGF-*β* release by tumors cells [33]. In addition, because interferon (IFN)*α* enhances IFN*γ* and IL-12 secretion, which in turn triggers NK cytotoxicity, Lion et al. proposed IFN*α* post-CR maintenance immunotherapy for AML patients [55].

Another therapeutic approach to enhance NK cell cytotoxicity is the use of cytokines, such as IL-2, IL12, and IL18 alone, or in association with histamine dihydrochloride, which enhance both NK- and T-lymphocyte proliferation and cytotoxicity [56, 57]. In AML, a postconsolidation regimen with an association between histamine dihydrochloride and IL-2 has resulted in improved progression-free survival (PFS) for patients (at 3 years PFS 40% *versus* 26% for controls) [58]. This association protected NK from the downregulation of activating-receptor expression (more particularly NKp46/NCR1) induced by leukemia cells; thus, it enhanced NK cytotoxicity and tumor immune control with significant improvement in leukemia-free survival [59].

Chemokine manipulation is also of interest as molecules may both attract NK to the tumor-cell microenvironment and stimulate their cytotoxic properties [60]. Specific chemokine inhibitors are currently under investigation, although redundancy and pleiotropy of the chemokine system are obstacles in drug development [61]. An alternative immunotherapy based on cytokine manipulation is NK stimulation via dendritic cells (DCs). Stimulation of DCs using TLR9 agonists converts tolerogenic DCs into immunogenic DCs, and allows DC activation and cytokine secretion, which enhances NK cytotoxicity [62].

Another attractive approach to enhance NK cytotoxicity is to use monoclonal antibodies (mAbs). IPH-2101, a fully human IgG4 anti-KIR mAb (developed by Innate Pharma) is currently being tested in phase I and II clinical trials in patients with AML and MM [63]. Its blockade of inhibition could allow NK-cell activation when activating ligands are present on target cells. Preliminary results show enhanced NK cell activity has a good safety profile [63]. Bispecific mAbs directed against both the target cells and cytotoxic effectors (NK cells) are also currently under investigation. Anti-CD20 mAbs that have enhanced affinity for CD16 have been also developed, and they are more effective at NK activation than rituximab [64, 65]. Similarly, bispecific mAbs, which targets the CD16 molecule on the NK cell surface and CD30 (the molecule expressed in Hodgkin's tumor and anaplastic T-cell lymphoma), are currently being evaluated [66].

Novel drugs with immunomodulatory properties are increasingly being used these days to treat hematological malignancies. Most have an impact on NK cell activity and/or target susceptibility to NK lysis.  Table 2 briefly outlines the major mechanisms identified or suspected that could explain their impact on NK cell cytotoxicity (see [9]).

## 6. Conclusion

 As the knowledge of NK cells' role in tumor surveillance increases, therapies to boost NK immunity have emerged. Because impairment of NK cell cytotoxicity is associated with almost all hematological malignancies, restoration of normal NK function is an attractive goal for immunotherapy. New approaches attempt to boost immune surveillance by enhancing NK cytotoxicity and tumor-cell susceptibility to NK lysis. Because the mechanisms that tumors use to escape NK surveillance are multiple, there are multiple potential ways to increase the NK cell lysis of tumor cells, that is, by increasing expression of activating receptors, diminishing or counteracting expression of inhibitory receptors, increasing NK cell cytotoxicity, and modulating target-cell sensibility to NK cell lysis via upregulation of ligand expression on the tumor-cell's surface. Combined strategies, including cytotoxic conventional chemotherapy with immunomodulatory agents, and NK cell manipulation, are becoming increasingly attractive.

## Figures and Tables

**Table 1:** Mechanisms of escape, receptor, and ligand involved and type of hematological malignancy.

Mechanisms of escape	Receptor in NK cells	Ligand in tumor cells	Hematological malignancy type	References
NK-cell quantitative deficiency			MDS	[5, 11]

Increased expression of inhibitory receptors		Upregulation of HLA class I	AML	[12–14]
			CLL LAL	[14, 15] [14]
			MM	[16]
			Lymphoma	[17]

Decreased activation by decreased expression of activating receptor or their ligands	NKp30		AML	[8, 18–20]
	NKp46NKG2D		CLL	[21]
		NCR-ligand	AML	
			LGL	[8]
		sMICA and sMICB	CLL	[22]
			CML	[23]
			MM	
			ALL	[24]
	DNAM1	NKG2D on tumor cells	CMML	[25]
				[26]
	CD94/NKG2C		AML	
				[27]
	2B4/CD244		AML	
				[27]
	CD16		AML	
			MM	[27]
			MM	[28, 29]
				[28, 29]

Impaired NK cell differentiation signaling			CML	[30]
			PV	Personal data

Impaired cytokine production	Elevated TNF		MDS	[31]
	Elevated PDGF		MPS	[32]
	Elevated TGFb			[33]
	Decreased IL1		AML	[34]
	IL2 and IFN*γ*		ALL	[34]

Abbreviations: MDS: myelodysplastic syndrome; MPS: myeloproliferative syndrome; ALL: acute lymphoid leukemia; AML: acute myeloid leukemia; CML: chronic myeloid leukemia; MM: multiple myeloma; CMML: myelomonocytic leukemia; LGL: large granular lymphoma; PV: polycythemia vera; sMICA: stress-induced molecule HLA class-I chain-related A; IL: interleukin; IFN*γ*: interferon-gamma.

**Table 2:** Novel agents used in hematological malignancies and their impact on NK-cell activation.

Class of drugs	Therapeutic molecules	Main indications	Inhibitory receptor ligands	Activating receptors	Activating receptor ligands	References
IMIDs	Thalidomide	Multiple myeloma		NKp46		[67]
	Lenalidomide			upregulation		

HDACI	Vorinostat	Lymphoma		NKp30 and NKp46	NKG2D and	[68, 69]
	Panobinostat				DNAM ligand	
				downregulation	upregulation	

Demethylating agents	5-azacytidine	MDS	Up regulation		NKG2D ligand	[70, 71]
	5-aza-2^′^-deoxycytidine		KIR		upregulation	

Proteasome inhibitors	Bortezomib	Multiple myeloma	Downregulation HLA molecules		NKG2D TRAIL and DNAM	[72–75]
					ligand upregulation	

All-trans retinoic acid	Vesanoid	AML3		NKG2D		[76]
				Upregulation		

Tyrosine kinase inhibitors	Imatinib mesylate	CML		NKG2D	sMICA	[77]
				upregulation	downregulation	

Abbreviations: IMIDs: immunomodulaory drugs; HDACI: Histone deacetylase inhibitors; MDS: myelodysplastic syndrome; AML: acute myeloid leukemia; CML: chronic myeloid leukemia; NK: natural killer cell; sMICA: stress-induced molecule HLA class-I chain-related A.
